# Erlotinib Treatment in Colorectal Cancer Suppresses Autophagy Based on *KRAS* Mutation

**DOI:** 10.3390/cimb46070447

**Published:** 2024-07-16

**Authors:** Alexander Siegman, Aaron Shaykevich, Danbee Chae, Isaac Silverman, Sanjay Goel, Radhashree Maitra

**Affiliations:** 1 Department of Biology, Yeshiva University, New York, NY 10033, USAsarah.chae@yu.edu (D.C.);; 2 Department of Medical Oncology, Rutgers Cancer Institute of New Jersey, New Brunswick, NJ 08901, USA

**Keywords:** erlotinib, KRAS, autophagy, colorectal, cancer, EGFR

## Abstract

The *KRAS* gene is mutated in approximately 45% of colorectal cancer patients. There are currently very few targeted treatments or therapies equipped to directly inhibit *KRAS* due to its unusual structural intricacies. Erlotinib, an EGFR inhibitor, has previously been demonstrated to reduce cell viability by inducing autophagy in lung cancer cell lines with varying EGFR mutations. In contrast to lung cancer cells, evidence is provided herein for the first time that erlotinib treatment in colorectal cancer (CRC) cell lines reduces autophagy and still results in decreased cell viability. However, the effects of erlotinib in CRC cell lines containing a wildtype *KRAS* gene were different than in cells carrying a mutant *KRAS* gene. We show that there is significantly more downregulation of autophagy in *KRAS* mutant CRC cells compared to *KRAS* wildtype cells, both at transcriptional and translational levels, suggesting that the *KRAS* mutation is advantageous for cancer growth, even in the presence of erlotinib. Cell viability results determined that *KRAS* wildtype CRC cells had significantly more cell death compared to *KRAS* mutant cells. Using patient mRNA datasets, we showed that there was a significant correlation between the presence of the *KRAS* mutation and the expression of autophagy proteins. Additionally, through molecular dynamics simulations, we develop a blueprint for *KRAS* and autophagy protein interaction and the impact of the *KRAS* mutation on autophagy protein regulation. Overall, this is the first report of erlotinib treatment in CRC cells that assesses autophagy, and we demonstrate that autophagy activity is downregulated in these cells. This effect is not only greater in cells carrying a *KRAS* mutation compared to wildtype cells, but the *KRAS* mutant cells also have increased cell viability compared to wildtype cells. We hypothesize that the difference in cell viability and autophagy expression between *KRAS* mutant and *KRAS* wildtype cells after treatment with erlotinib can be of therapeutic value to treat CRC patients carrying *KRAS* mutations.

## 1. Introduction

In 2023, colorectal cancer (CRC) was the second largest cause of cancer death in the USA, with over 52,000 deaths [1]. This is equivalent to 9% of all deaths caused by cancer in the USA [1]. The *KRAS* mutation (*KRAS*-mut) is prevalent in approximately 45% of the total cases of CRC [2]. The adenomatous polyposis coli (*APC*) is a prominent tumor suppression gene; however, the *APC* gene is frequently mutated and promotes CRC onset and development [3,4,5].

There are many different *KRAS* mutations found in cancer patients, including G12D (33.7%), G12V (32.7%), G12C (14%), and G13D (12.5%) [6,7,8]. The G12D and G12V mutations already have small-molecule inhibitors that are being tested in the clinic. The G13D mutation does not yet have that and was, therefore, our focus. 

Autophagy is a systematic cellular process that utilizes lysosomes to digest and break down various substances within the cell, such as proteins and microorganisms, which are reused to provide nutrients and similar necessities to the cells [9]. However, if untimely cellular malfunctions or mutations occur within the cells, autophagy may be induced, which, in turn, can lead to the development of different cancers [10,11]. It has been reported that autophagy protein activity, for example, LC3II expression and ATG5/ATG12 conjugation, were upregulated in lung cancer cells [10].

Erlotinib is a small molecule inhibitor of the epidermal growth factor receptor (EGFR), which is a transmembrane receptor tyrosine kinase (RTK) necessary for cell proliferation through downstream signaling [12,13]. Although different from our results, erlotinib has been previously demonstrated to induce autophagy in lung cancer cells [14]. Erlotinib inhibits metabolic activity in CRC cells and induces apoptosis [15]. The most common acquired resistance mechanism that first-generation cells experience after treatment with erlotinib is the emergence of the T790M gatekeeper mutation in the ATP binding pocket of EGFR [16]. Although erlotinib has been previously explored on CRC, the impacts of autophagy and the *KRAS* mutation remain unknown, which is why it is important to further experiment on CRC with erlotinib. Experiments have previously demonstrated that patients can develop resistance to erlotinib due to its nature as a competitive inhibitor seen in lung cancer [17]. Furthermore, treatment with chloroquine on lung cancer has proven useful for synergetic treatment alongside erlotinib due to chloroquine’s effects as an autophagy inhibitor [18]. This would provide valuable insight into the therapeutic viability of the regulation of autophagy to increase the efficacy of erlotinib in *KRAS*-mut CRC cells that lack a well-defined line of treatment. Furthermore, understanding the different effects of erlotinib on *KRAS*-mut and *KRAS*-wildtype (WT) CRC can help guide synergistic treatments in *KRAS*-mut CRC with erlotinib. 

## 2. Materials and Methods

### 2.1. Cell Lines

This study employed two CRC cell lines, namely HCT116 and Hke3. HCT116 is characterized by a G13D *KRAS* mutation, while Hke3 is a *KRAS*-WT isogenic derivative of HCT116 [14]. The HCT116 cell line was procured from the American Type Culture Collection (ATCC^®^, Manassas, VA, USA), and the Hke3 cell line was obtained from Dr. Takehiko Sasazuki at the Medical Institute of Bioregulation, Kyushu University.

### 2.2. Cell Culture

Cell lines were cultured in RPMI 1640 media (Gibco™, Grand Island, NY, USA, Catalog #: 11875093), supplemented with 10% Fetal Bovine Serum (GemCell™, West Sacramento, CA, USA, Catalog #: 100-500), 1% Non-Essential Amino Acids (Gibco™, Catalog #: 11140050), 2% HEPES buffer (Gibco™, Catalog #: 15630080), 1% Anti-Anti (Gibco™, Catalog #: 15240062), and 0.4% gentamicin (Gibco™, Catalog #: 15710064). The cells were nurtured in an environment containing 5% CO_2_ at 37 °C and were passaged following the recommended protocol of ATCC^®^.

### 2.3. Erlotinib Preparation

The erlotinib (Cayman Chemical^TM^, Ann Arbor, MI, USA, Item #: 10483) powder was dissolved in DMSO at a concentration of 2 mg/mL and placed into single-use aliquots at −20 °C. 

### 2.4. Cell Viability Assay

HCT116 and HKe3 cells were cultured until 70% confluency, at which point, they were treated with trypsin and counted using the Countess™ II, Automated Cell Counter (Invitrogen™, Waltham, MA, USA, Catalog #: AMQAX1000) with Trypan Blue solution (Sigma-Aldrich™, Burlington, MA, USA, Catalog #: T8154) as per the manufacturer’s protocol. Dilutions were made so that each well of a BrandTech^®^cellGrade™ 96-well plate (BrandTech^®^, Essex, CT, USA, Catalog #: 781968) had 20,000 cells in 90 μL of RPMI. The pipetting was performed using a PIPETMAN M Multichannel P12x1200M (Gilson™, Middleton, WI, USA, Catalog #: F81021) pipette. The 96-well plates were then placed in an incubator with an atmosphere of 5% CO_2_ at 37 °C for 24 h.

Twenty-four hours later, 10 μL of 350 μM and 200 μM of an erlotinib solution (originally dissolved in DMSO) diluted in RPMI were added to different columns of the 96-well plate to make concentrations of 35 μM and 20 μM, respectively. A wide range of concentrations were tested but consistency was noted above 10 μM. Hence, we decided to use 20 μM for our in vitro experiments. The DMSO percentage in the 35 μM and 20 μM concentrations of erlotinib are 0.194% DMSO and 0.111% DMSO, respectively. A separate column in the 96-well plate was reserved as a control for the highest concentration of DMSO, which was 0.194%, determined from the 35 μM treated well. DMSO showed no effect on toxicity. The 96-well plates were then placed in an incubator with an atmosphere of 5% CO_2_ at 37 °C for 9 h, 24 h, and 48 h.

After 9 h, 24 h, or 48 h, 10 μL of Invitrogen™ PrestoBlue™ HS Cell Viability Reagent (Invitrogen™, Waltham, MA, USA, Catalog #: P50200) was added to all the wells of the 96-well plates using a PIPETMAN M Multichannel P12x200M (Gilson™, Catalog #: F81030) pipette. The 96-well plates were then placed in an incubator with an atmosphere of 5% CO_2_ at 37 °C for 1 h. Beyond 48 h, the treated cells died rapidly.

One hour later, the absorbance readings were measured of the 96-well plates using SpectraMax Mini Multi-mode Microplate reader following the PrestoBlue™ manufacturer protocol to determine its values. 

The percent change between the control and treated cells was calculated by normalizing the treated cells with the untreated control cells. All reactions were prepared in octuplets using all eight wells per column for every experimental data point.

### 2.5. Gene Expression Profiling Interactive Analysis 2 (GEPIA2)

The GEPIA2 online website was used to analyze the correlation of genes in CRC patients from The Cancer Genome Atlas (TCGA) database [19]. We assessed the correlation between gene expression of *ATG5*, *MAP1LC3B*, *ATG10*, *ATG13*, and *ATG14* with the *KRAS* mutation. This was conducted on the GEPIA2 website by selecting the Multiple Gene Analysis tab, selecting Correlation Analysis, and then selecting the following options: Gene A = relevant autophagy gene, Gene B = *KRAS*, Normalized by gene = TUBA1A, Correlation Coefficient = Spearman, and Used Expression Datasets = COAD Tumor & READ Tumor, and then separately selected Used Expression Datasets = COAD Normal & READ Normal.

### 2.6. TCGA Gene Expression

To assess gene expression in CRC patients from the TCGA database, the UCSC Xena online website was utilized [20]. The analysis involved selecting the three dots above the box containing the three subgroups of samples and opting for differential expressions. For the initial mRNA change calculation in cancer, subgroup A encompassed both cancer groups, while subgroup B included the Solid Tissue control. Subsequently, for the evaluation of mRNA changes in the presence of a *KRAS* mutation, subgroup A comprised *KRAS*-mut data, and subgroup B incorporated *KRAS*-wildtype data. The advanced settings remained unaltered. A file providing Log(2)FC, *p*-value, and adjusted *p*-value was obtained, and the fold change of the target gene was computed.

To visualize these changes, the mRNA expression of the target genes was accessed. The “view as chart” symbol was chosen, and “compare subgroups” was then selected. “Show data from” included the target gene, and “subgroup samples by” included our three groups of samples.

### 2.7. Protein Extraction

To prepare the Cell Extraction Buffer (Invitrogen^TM^, Catalog #: FNN0011), 10 µL of Thermo Scientific™ Halt™ Protease and Phosphatase Inhibitor Cocktail EDTA-free (100X) (Thermo Scientific™, Waltham, MA, USA, Catalog #: 78445) was added to a microcentrifuge tube for every 1 mL of freeze/thaw lysis buffer. Subsequently, each cell pellet was resuspended in 250 µL of the freeze/thaw lysis buffer containing protease and phosphatase inhibitor. The cell pellets underwent three cycles of brief immersion in liquid nitrogen for 10 s, followed by thawing and vertexing. After the third freezing cycle with liquid nitrogen, cells were placed on ice to thaw. The thawed cell pellets were then subjected to centrifugation at maximum speed for 45 min at 4 °C, and the resulting supernatants were aliquoted into single-use containers and stored at −80 °C.

### 2.8. Protein Quantification

Protein quantification was conducted through a Bradford assay, wherein each 1.5 mL tube contained a mixture of 500 μL of H_2_O, 495 μL of Bradford reagent, and 5 μL of either the protein sample or BSA standard (with an additional 5 μL of Bradford for the plate blank). Subsequently, 600 μL of this composite was dispensed into 3 wells of a 96-well plate (Corning™, Corning, NY, USA, Catalog #: 3598) at 200 μL per well. The plate was then analyzed using a SpectraMax Mini multi-mode microplate reader (Molecular Devices™, Silicon Valley, USA, Catalog #: 76640-506) to measure absorbance at 595 nm. All readings were corrected by subtracting the plate blank, and the triplicate values were averaged. A standard curve, generated using BSA standards, facilitated the estimation of protein concentrations based on this curve.

### 2.9. Western Blot Analysis

For each sample, 40 μg of protein was used. Protein preparation involved mixing 1 part protein with 1 part 2x Lammeli sample buffer (Bio-Rad™, Hercules, CA, USA, Catalog #: 1610737) and subjecting the mixture to boiling water for 10 min. Loading was performed on a 4–20% gel (Bio-Rad™, Catalog #: 4561094), incorporating 2 μL of Magic Marker (Invitrogen™, Catalog #: LC5602) and 10 μL of protein ladder (Thermo Scientific™, Catalog #: 26616). Transfer to a nitrocellulose membrane (Bio-Rad™, Catalog #: 1620215) was accomplished through a wet transfer at 80 mV for 1 h.

After the transfer, membranes underwent blocking in 1% BSA in TBS for one hour, followed by overnight exposure to antibodies. The primary antibody for LC3A was Invitrogen™ LC3A Monoclonal Antibody (7F5R1) at a 1:1000 dilution (Invitrogen™, Catalog #: MA5-35164). For ULK1, the primary antibody used was Invitrogen™ ULK1 Recombinant Rabbit Monoclonal Antibody (JA58-36) at a 1:1000 dilution (Invitrogen™, Catalog #: MA5-32699). For ATG5, the primary antibody used was Protein Tech™ ATG5 Mouse Monoclonal Antibody (Protein Tech^TM^, Manchester, UK, 66744-1) at a 1:3000 dilution (Invitrogen™, Catalog #: MA5-32699). For Beclin-1, the primary antibody used was Protein Tech™ Beclin-1 Mouse Monoclonal Antibody (66665-1) at a 1:3000 dilution (Invitrogen™, Catalog #: MA5-32699). Beta-actin detection employed Abnova ACTB Monoclonal Antibody (M01) clone 3G4-F9 at a 1:750 dilution (Abnova, Catalog #: H00000060-M01).

To determine the expression of the autophagy proteins ATG5, LC3A, ULK1, and Beclin-1, the cells were treated with 20 μM of erlotinib. The concentration was chosen through experimentation with varying concentrations, as well as finding 20 μM being previously used in CRC experiments involving autophagy expression [15,21]. After 48 h of treatment, the CRC cells were harvested, and the protein was isolated and quantified.

Detection of antibodies was achieved using the Pierce™ Fast Western Blot Kit (Thermo Scientific™, Catalog #: 35050), following the manufacturer’s protocol. The ChemiDoc MP imaging system (Bio Rad™, Catalog #: 1708280) was employed for chemiluminescence detection.

### 2.10. RNA Extraction and Quantification

Cell pellets were subjected to RNA extraction employing the Invitrogen™ PureLink™ RNA Mini Kit (Invitrogen™, Carlsbad, CA, USA, Catalog #: 12183018A) following the manufacturer’s protocol. The resulting purified RNA was then divided into single-use aliquots and stored at −80 °C. Quantification of the extracted RNA was conducted using a Thermo Scientific™ NanoDrop 1000 (Thermo Scientific™, Catalog #: 2353-30-0010). Assessment of the RNA’s quality involved checking the 260/280 ratio, and only RNA samples within the range of 1.9 to 2.1 were retained.

### 2.11. cDNA Synthesis

The extracted and quantified RNA, amounting to 1.5 μg, was then synthesized to cDNA utilizing the iScript Reverse Transcription Supermix for RT-qPCR (Bio-Rad™, Catalog #: 1708841), following the manufacturer’s protocol. The reaction was conducted on a T100™ Thermal Cycler (Bio-Rad™, Catalog #: 1861096). Subsequently, 100 μL of autoclaved water was added to each sample. The synthesized cDNA was evaluated using the Thermo Scientific™ NanoDrop 1000 and then diluted to a concentration of 25 ng/μL with DEPC-treated water. The resulting cDNA was portioned into single-use aliquots and stored at −80 °C.

### 2.12. Quantitative Polymerase Chain Reaction (qPCR)

Primers, procured from Sigma-Aldrich™ (Easy Oligo), were received pre-diluted in deionized water at a 100 µM concentration. Upon arrival, the primers were aliquoted for single-use and stored at −20 °C. Refer to Table 1 for the primer sequences.

For qPCR preparation, 10 µL of forward primer, 10 µL of reverse primer, and 180 µL of Thermo Scientific™ DEPC treated water (Thermo Scientific, Catalog #: FERR0601) were combined to achieve a 5 µM final concentration of both forward and reverse primers. Each qPCR well received 1 µL of the prepared 5 µM primer mix, 4 µL of synthesized cDNA, and 5 µL of Applied Biosystems™ PowerUp™ SYBR™ Green Master Mix (Applied Biosystems™, Foster City, CA, USA, Catalog #: A25918), resulting in a final reaction mix with a 500 nM concentration for each primer and 100 ng of cDNA. The qPCR reactions were run using a Quantabio™ Q cycler (Quantabio™, Beverly, MA, USA, Catalog #: 95900-4C), with all reactions prepared in triplicate.

Data analysis employed the ΔΔCT method, where ΔCT was initially determined by subtracting the GAPDH CT value of each sample from its average target gene CT value. Subsequently, ΔΔCT was calculated by subtracting the ΔCT of the untreated cell from the ΔCT of the treated cell. The delta–delta CT values were then transformed into fold change values using 2^(−ΔΔCT)^.

The analysis of mRNA was performed by treating the HCT116 and HKe3 cells with 20 μM of erlotinib. After 48 h of treatment, the RNA was harvested, and the resulting cDNA was synthesized.

### 2.13. Protein–Drug Docking

The AI AlphaFold predicted structure for human EGFR (ID: AF-P00533-F1), https://www.uniprot.org/uniprotkb/P00533/entry (accessed on 14 February 2024), was downloaded from the Uniprot database in Protein Data Bank (PDB) format [22,23]. The model structure for erlotinib (ID: AQ4), https://www.rcsb.org/ligand/AQ4 (accessed on 14 February 2024), was downloaded from the RCSB database. Using the CB-Dock2 server, https://cadd.labshare.cn/cb-dock2/index.php (accessed on 14 February 2024), erlotinib was successfully docked to EGFR in silico [24,25]. Five suitable binding conformations were generated by the CB-Dock2 server and ranked in order of structural and energetic favorability. The highest-ranked conformation was chosen to be analyzed.

### 2.14. Protein–Protein Molecular Dynamics Simulation and Kinetic and Structural Analysis

The AlphaFold AI predicted pdb files for Human *KRAS* and Human ATG5 were downloaded from Uniprot in PDB format [22,23]. PyMOL software (Version 2.0 Schrodinger, LLC, New York, NY, USA) was used to induce a mutation in the *KRAS* file to generate the G13D mutant. *KRAS*-WT and the G13D mutant were individually docked to ATG5 using the ClusPro server, which generated the most energetically favorable binding conformations of the two proteins [26,27,28,29]. ATG5 was set as the receptor (Chain A), and *KRAS*-WT and *KRAS*-mut (G13D) were set as the ligand (Chain B). The most energetically favorable dockings were chosen, and using GROMACS (Version 2020), a Linux-based molecular dynamic simulation software, topology files were generated for each complex. The complexes were fixed in a cubic cell environment, which was solvated with water and ionized for neutral charge. An energy minimization was performed for the topologies to prevent any steric collisions and set an optimal starting point for simulation. Temperature and pressure were each equilibrated before a 10 ns simulation was performed. 

gmx_MMPBSA energy calculation software (Version gmx_MMPBSA v1.5.1) was used for kinetic analysis [30]. Per-residue (residue–complex) and Per-wise (residue–residue) analyses were performed. Per-residue provided binding interaction energy data about an individual residue and the entire complex, while Per-wise provided binding interaction energy data about individual residues with other individual residues. Total complex energy (bound state energy), receptor and ligand energies (unbound state energy), and delta energy (delta energy = bound energy − unbound energy) were calculated by gmx_MMPBSA. Total complex energy indicated overall complex kinetic stability, and delta energy indicated kinetic binding strength. gmx_MMPBSA generated line plots, heat maps, and associated value tables depicting the energy values used for analysis. Only residues that contributed >0.0100 kcal/mol of binding energy were used for analysis. 

Root mean square fluctuation (RMSF), root mean square deviation (RMSD), and radius of gyration (Rg) were calculated using GROMACS and plotted using XmGrace software (Version Grace-5.1.22) for structural analysis. RMSF plots the average fluctuation of individual residues of each protein from their original position throughout the simulation. RMSD plots the overall backbone fluctuation against time throughout the simulation. Rg plots the change in protein conformation, specifically how dense the protein is.

### 2.15. Statistical Analysis

Statistical analysis was performed using Microsoft™ Office Excel (https://www.microsoft.com/en-us/microsoft-365/excel, accessed on 14 January 2024). The bar and line graphs were made using Excel. The statistical significance (*p* < 0.05) was determined using a two-tailed *t*-test. Outliers were determined and removed based on Iglewicz and Hoaglin’s outlier test with modified z-scores using the recommended outlier criterion of modified Z score ≥ 3.5.

## 3. Results

### 3.1. Erlotinib Induces Greater Cell Killing in KRAS Wildtype Hke3 Compared to KRAS Mutant HCT116 Cells

**Figure 1:**
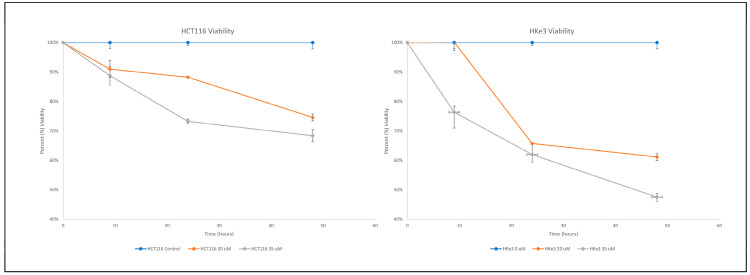
Four experiments were averaged for all drug concentrations at each time point. Each experiment was performed with erlotinib treatment of 20 μM and 35 μM. HCT116 at 9 h for treatments of 20 μM and 35 μM had a significance of *p* < 0.05 and 0.05, respectively. HCT116 at 24 h with treatments of 20 μM and 35 μM had a significance of *p* < 0.05 and 0.001, respectively. HCT116 at 48 h with treatments of 20 μM and 35 μM had a significance of *p* < 0.05 and 0.01, respectively. HKe3 at 9 h with treatments of 20 μM and 35 μM had a significance of *p* > 0.05 and *p* < 0.05, respectively. HKe3 at 24 h with treatments of 20 μM and 35 μM had a significance of *p* < 0.05 and 0.05. HKe3 at 48 h with treatments of 20 μM and 35 μM had a significance of *p* < 0.001 and 0.001, respectively. There was greater cell death in HKe3 compared to HCT116 at 9 h for 20 μM and 35 μM, with a significance of *p* < 0.05 for all. At 24 and 48 h at the same concentrations, there was a significance of *p* < 0.05 and *p* > 0.05, respectively.

Assessment of viability of HKe3 and HCT116 cell lines after treatment with erlotinib was performed at three different time points (9 h, 24 h, and 48 h) and two concentrations of erlotinib (20 μM and 35 μM). For HKe3, 9 h of treatment was non-significant at 20 μM but significant (*p* < 0.05) at 35 μM. At 24 h of treatment for HKe3, there was significance at both concentrations of erlotinib (*p* < 0.05). For 48 h of treatment, there was a significance (*p* < 0.001) for all HKe3 readings. For HCT116, 9 h, 24 h, as well as 48 h of treatment demonstrated a significance (*p* < 0.05) at both concentrations. When the altered cell viabilities between the two cell two lines were compared, *KRAS*-WT CRC (Hke3) experienced significantly greater cell death compared to *KRAS*-mut CRC (HCT116) at all time points (*p* < 0.05) (Table 2), except for a 9-h treatment at 20 μM in which HCT116 had significantly greater cell death, as well as a 24-h treatment at 35 μM in which there was no statistically significant experimental difference (Figure 1).

### 3.2. The Expression of Key Autophagy Proteins Decreases in KRAS-Mut upon Treatment with Erlotinib When Compared to KRAS-WT

**Figure 2:**
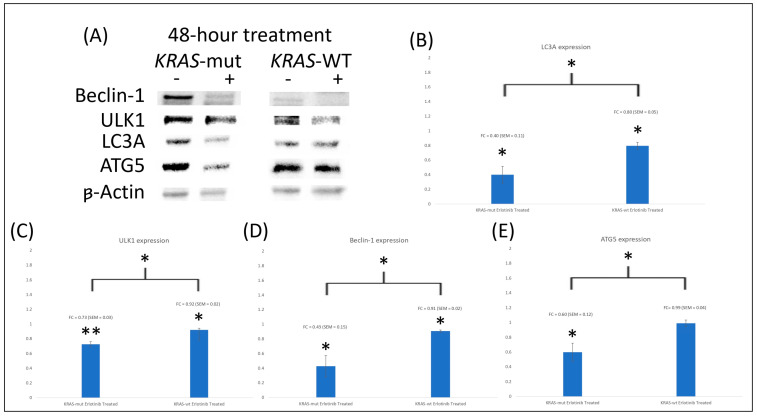
Four experiments were averaged for all proteins at 48 h of treatment. The symbol * represents significance of *p* < 0.05, ** represents significance of *p* < 0.01. (**A**) Beclin-1, ULK1, LC3A, and ATG5 were downregulated in both *KRAS*-WT and *KRAS*-mut. All the proteins were more significantly downregulated in *KRAS*-mut compared to *KRAS*-WT (*p* < 0.05). (**B**) Densitometric analysis of LC3A was averaged, and the mean and standard deviation were calculated. Protein expression of LC3A was significantly downregulated in *KRAS*-mut and *KRAS*-WT (*p* < 0.05). *KRAS*-mut was significantly more downregulated compared to *KRAS*-WT (*p* < 0.05). (**C**) Densitometric analysis of ULK1 was averaged, and the mean and standard deviation were calculated. Protein expression of ULK1 was significantly downregulated in *KRAS*-mut and *KRAS*-WT (*p* < 0.01, 0.05, respectively). *KRAS*-mut was significantly more downregulated compared to *KRAS*-WT (*p* < 0.05). (**D**) Densitometric analysis of Beclin-1 was averaged, and the mean and standard deviation were calculated. Protein expression of Beclin-1 was significantly downregulated in *KRAS*-mut and *KRAS*-WT (*p* < 0.05). *KRAS*-mut was significantly more downregulated compared to *KRAS*-WT (*p* < 0.05). (**E**) Densitometric analysis of ATG5 was averaged, and the mean and standard deviation were calculated. Protein expression of ATG5 was significantly downregulated in *KRAS*-mut (*p* < 0.05) but was non-significant in *KRAS*-WT. *KRAS*-mut was significantly more downregulated compared to *KRAS*-WT (*p* < 0.05).

Our data demonstrate that there was significant downregulation (*p* < 0.05) for all autophagy proteins in both HCT116 and HKe3 (representative Western blot is shown in Figure 2A), except for ATG5 expression in HKe3 (Figure 2E), which was non-significant. The fold change for all the proteins is shown in Figure 2B–E. The autophagy proteins ATG5, Beclin-1, LC3A, and ULK1 are significantly (*p* < 0.05) more downregulated in *KRAS*-mut than in *KRAS*-WT cells. This demonstrates that treatment with erlotinib results in the downregulation of autophagy proteins in *KRAS*-mut CRC compared to *KRAS*-WT CRC cell lines. The fold changes are shown in Table 3 below. There was significantly (*p* < 0.05) more downregulation of autophagy proteins in HCT116 when compared to HKe3. 

### 3.3. ULK1, BECN1, ATG5, and MAP1LC3B mRNA Expression Is Reduced in KRAS-mt CRC Compared to KRAS-WT CRC Cells

**Figure 3:**
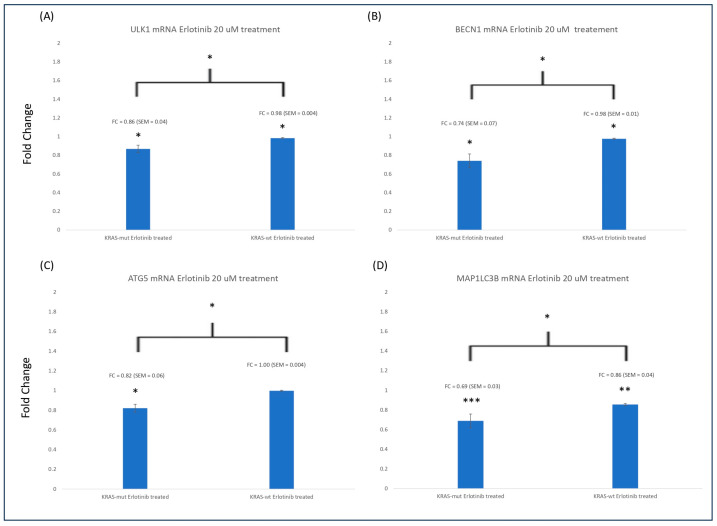
Four experiments were averaged for all genes at 48 h of treatment. The symbol * represents significance of *p* < 0.05, ** represents significance of *p* < 0.01, and *** represents significance of *p* < 0.001. (**A**) ULK1 mRNA expression was significantly downregulated in *KRAS*-mut and *KRAS*-WT (*p* < 0.05). *KRAS*-mut was significantly more downregulated compared to *KRAS*-WT (*p* < 0.05). (**B**) BECN1 mRNA expression was significantly downregulated in *KRAS*-mut and *KRAS*-WT (*p* < 0.05). *KRAS*-mut was significantly more downregulated compared to *KRAS*-WT (*p* < 0.05). (**C**) ATG5 mRNA expression was significantly downregulated in *KRAS*-mut (*p* < 0.05) but not significant in *KRAS*-WT. *KRAS*-mut was significantly more downregulated compared to *KRAS*-WT (*p* < 0.05). (**D**) MAP1LC3B mRNA expression was significantly downregulated in *KRAS*-mut and *KRAS*-WT (*p* < 0.001, 0.01, respectively). *KRAS*-mut was significantly more downregulated compared to *KRAS*-WT (*p* < 0.05).

There was significant downregulation (*p* < 0.05) of ULK1, BECN1, ATG5, and MAP1LC3A mRNA for both *KRAS*-WT CRC and *KRAS*-mut CRC for all values except ATG5 in *KRAS*-WT which was not significant (Figure 3A–D). For all autophagy-related genes, the mRNA was significantly (*p* < 0.05) downregulated in *KRAS*-mut compared to *KRAS*-WT (Figure 3). This demonstrates that the effects of erlotinib in *KRAS*-mut are much greater than in *KRAS*-WT cells. The fold changes between cell lines are shown in Table 4. We found that there was significantly (*p* < 0.05) more downregulation of autophagy genes in HCT116 compared to HKe3 cells. 

### 3.4. KRAS Expression Positively Correlates with the Expression of the Autophagy Genes MAP1LC3B, ATG5, ATG10, ATG13, and ATG14 in CRC Patients

**Figure 4:**
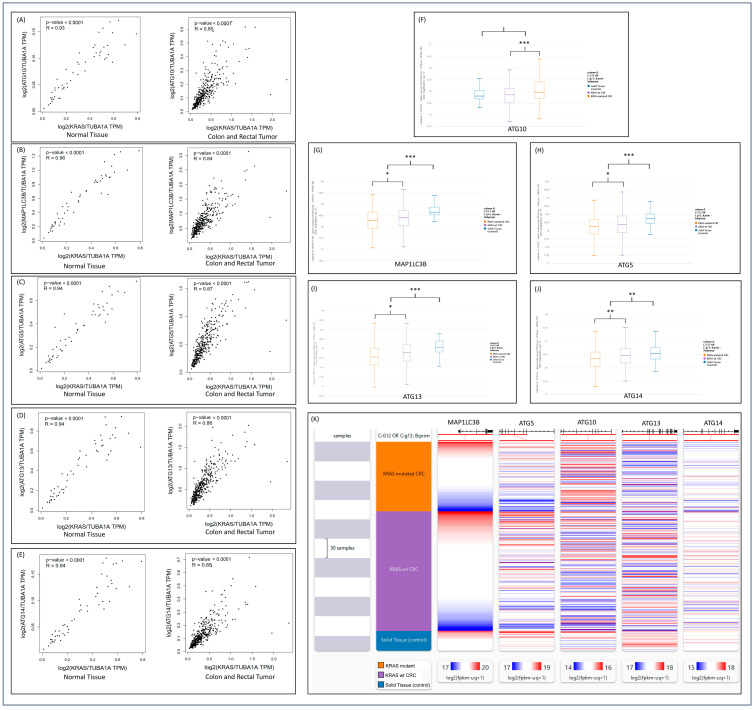
The symbol * represents significance of *p* < 0.05, ** represents significance of *p* < 0.01, and *** represents significance of *p* < 0.001. (**A**) Positive correlation between *KRAS* and ATG10 in normal tissue and colon or rectal tumors (*p* < 0.0001). (**B**) Positive correlation between *KRAS* and MAP1LC3B in normal tissue and colon or rectal tumors (*p* < 0.0001). (**C**) Positive correlation between *KRAS* and ATG5 in normal tissue and colon or rectal tumors (*p* < 0.0001). (**D**) Positive correlation between *KRAS* and ATG13 in normal tissue and colon or rectal tumors (*p* < 0.0001). (**E**) Positive correlation between *KRAS* and ATG14 in normal tissue and colon or rectal tumors (*p* < 0.0001). (**F**) ATG10 mRNA expression is further increased in *KRAS*-mutated CRC in patient datasets. (**G**) MAP1LC3A mRNA expression is further decreased in *KRAS*-mutated CRC in patient datasets. Expression of mRNA is also increased in Solid Tissue Control. (**H**) ATG5 mRNA expression is further decreased in *KRAS*-mutated CRC in patient datasets. Expression of mRNA is also increased in Solid Tissue Control. (**I**) ATG13 mRNA expression is further decreased in *KRAS*-mutated CRC inpatient datasets. Expression of mRNA is also increased in Solid Tissue Control. (**J**) ATG14 mRNA expression is further decreased in *KRAS*-mutated CRC inpatient datasets. Expression of mRNA is also increased in Solid Tissue Control. (**K**) A visual representation of the mRNA expression of MAP1LC3B, ATG5, ATG10, ATG13, and ATG14 in differing tissue types. Darker lines represent a higher expression.

In the process of gaining a clearer understanding of the crosstalk between the most prominent autophagy genes and the *KRAS* expression status without erlotinib treatment, correlation analysis was performed using GEPIA2 (http://gepia2.cancer-pku.cn/) accessed on 20 November 2023. We analyzed the correlation between *KRAS* mutational status and expression of several autophagy genes, including MAP1LC3B, ATG5, ATG10, ATG13, and ATG14 in both COAD (colon adenocarcinoma) READ (rectum adenocarcinoma) as well as normal tissue (Table 5). In both the control and cancer patients, there were positive correlations (*p* < 0.0001) between *KRAS* mutational status and the expression of MAP1LC3B, ATG5, ATG10, ATG13, and ATG14 (Figure 4). The difference in gene expression of normal tissue vs. colorectal cancer data was next utilized to revalidate our findings using UCSC Xena software (https://xena.ucsc.edu/, accessed on 6 December 2023) (Table 6). We reconfirmed the correlational analysis data with the expression of MAP1LC3B, ATG5, ATG13, and ATG14 in cancer patients, in *KRAS*-mut compared to *KRAS*-WT (*p* < 0.05) (Figure 4F–J). In Xena, however, ATG10 was found to be non-significantly overexpressed in cancer patients, although *KRAS*-mut expressed ATG10 more than *KRAS*-WT patients. The gene expression was expressed visually using Xena (Figure 4K).

### 3.5. Destabilization of KRAS-ATG5 Complex when KRAS Is Mutated (G13D)

**Figure 5:**
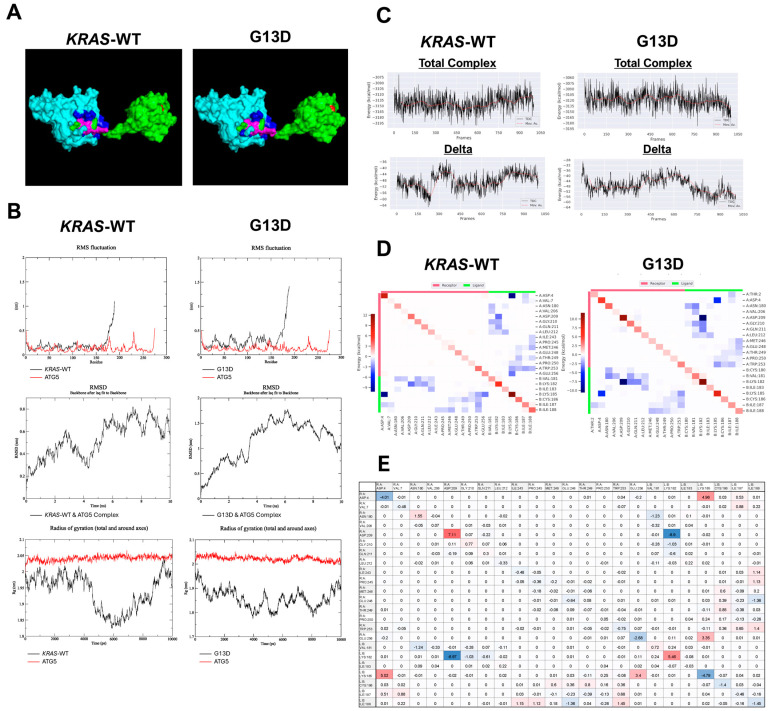
(**A**) 3D molecular model of the *KRAS*-WT and ATG5 and G13D and ATG5 complexes. *KRAS*-WT and G13D are colored green, and their delta residues of interaction are colored pink. ATG5 is colored cyan, and its delta residues of interaction are colored dark blue. The G13 residue is colored red. (**B**) RMSF, RMSD, and Rg plots describe complex structure and stability in each complex. (**C**) Per-residue total complex and delta energy line plots indicating complex stability and total binding strength throughout the 10 ns simulations of each complex. (**D**) Per-wise heat tables of individual residues–residue interactions for each complex. (**E**) Quantitative delta values between individual residue–residue interactions involved in the WT complex and those interactions in the G13D complex. Any residue–residue interactions that were present in the G13D complex but not the WT complex were removed to focus on the common residue–residue interactions that were present in both complexes. Additionally, any residue–residue interactions that were present in the WT complex but not the G13D complex were assigned a value of 0 kcal/mol to allow for delta energy to be calculated between the WT and G13D complexes. A more stable residue–residue interaction is defined by a larger negative energy value. Interactions shaded red were stronger (more negative) in the WT complex, and those shaded blue were stronger in the G13D complex. Interactions near 0 kcal/mol had little alteration between complexes.

The stability and binding affinity of the *KRAS*-ATG5 complex were determined using *KRAS*-WT and the G13D mutant cell lines by individually binding the *KRAS* and ATG5 proteins, followed by stimulation with ATG5 (Figure 5A). The overall effect of the G13D mutation on the interaction between the *KRAS* and ATG5 proteins was analyzed structurally and kinetically.

The most energetically favorable binding site of *KRAS* and ATG5 occurred at the c-terminus hypervariable region of *KRAS* and primarily at the c-terminus of ATG5, although a few significant interactions also did occur at the n-terminus and toward the center of the chain. Root mean square fluctuation (RMSF), root mean square deviation (RMSD), and radius of gyration (Rg) plots were generated to analyze structural changes between the WT and mutant complexes (Figure 5B). Plots of the mean square fluctuation calculations of the two proteins signified these segments as the sites for interactions in both the WT and mutant complex. Notably, there was an increase of approximately 0.25 nm in the c-terminus fluctuation of the G13D mutant when binding with ATG5 compared to WT. Furthermore, the n-terminal catalytic domain of the G13D experienced an increase in fluctuation compared to WT, notably including the Switch I and Switch II regions. Root mean square deviation plots for the WT and mutant complexes further supported a significant loss of complex stability when the mutation was induced. The RMSD of the mutant complex doubled compared to the WT complex. The WT complex experienced a maximum backbone fluctuation of approximately 0.8 nm and equilibrated between 0.6 nm and 0.8 nm. However, the mutant complex reached a maximum backbone fluctuation of approximately 1.75 nm and equilibrated between 1 nm and 1.25 nm. The radius of gyration plots for each complex additionally demonstrated an alteration in structural conformation of the G13D mutant compared to *KRAS*-WT. The radius of gyration for the G13D mutant was significantly decreased, indicating an increase in protein density. Although *KRAS*-WT experienced a dip of ~0.1 nm between 4 ns and 9 ns, the general equilibration amplitude of the complex occurred between 2.03 nm and 1.95 nm. Conversely, the G13D mutant experienced a broader equilibration amplitude between 1.95 nm and 1.78 nm throughout the simulation, demonstrating greater density fluctuations.

Per-residue energy calculations provided the kinetic stability and binding affinity strength of the complexes, using total complex and delta energies, respectively (Table 7) (Figure 5C). The calculated mean total complex energy for the WT complex was −23.6 kcal/mol less than the G13D complex, indicating higher stability in the WT complex. The standard deviation for the total complex energy was not significantly altered by the mutation. The delta mean of the WT increased by 0.7 kcal/mol, indicating little overall decrease in binding strength. Additionally, the standard deviation for the delta was not significantly altered by the mutation. 

Although the overall total complex binding strength did not appear to be significantly altered, Per-wise energy calculations revealed that the binding strength of numerous residues involved in the interaction had changed. The individual residue–residue interactions of the residues that contributed to the delta energy were calculated and plotted for the WT and G13D complexes (Figure 5D). Internal atomic interaction in individual residues composes the diagonal border of symmetry. Respectively, 24 and 22 residues were involved in the delta energy interactions, exhibiting bound and unbound energies. VAL^7^, ILE^243^, PRO^245^, and GLU^256^ of ATG5 exhibited delta energy interactions in the WT complex but not the G13D complex. THR^2^ of ATG5 and CYS^180^ of *KRAS* exhibited delta energy interactions in the G13D complex but not the WT complex. 

A delta calculation was performed for the residues involved in the WT interaction and the ones in the G13D interaction (Figure 5E). Residue–residue interactions only present in the G13D complex but not in the WT complex were removed to focus on the common residue–residue interactions that were present in both complexes. Additionally, residues present in the WT complex but not the G13D complex were added with a value of 0 kcal/mol to allow for delta energy to be calculated between the WT and G13D complexes. A more stable residue–residue interaction is defined by a larger negative energy value. Thus, here the effect of the mutation on the stability of specific residue–residue interactions is quantified. Interactions shaded red indicate that the WT complex residues had a stronger interaction energy (more negative) than the G13D complex residues. Interactions shaded blue indicate a stronger interaction in the G13D complex. Values closer to 0 kcal/mol indicate little change in binding strength between the complexes. Notably, along the diagonal border of internal atomic interactions, several residues experienced shifts toward having stronger energy contributions in one of the complexes. In *KRAS*, VAL^181^ and LYS^182^ exhibited a trend to participate more strongly in G13D complex interactions, except in their internal atomic interactions. LYS^182^’s interaction with ASP^209^ of ATG5 exhibited this trend as the strongest of all residues involved in the delta energy interaction. In *KRAS*, LYS^185^, CYS^186^, ILE^187^, and ILE^188^ exhibited a trend to participate more strongly in WT complex interactions, except in their internal atomic interactions. LYS^185^’s interactions with ASP^4^ and GLU^256^ of ATG5 exhibited this trend as the strongest of all residues involved in the delta energy interaction. ILE^187^ and ILE^188^ exhibited a few interactions that contradicted this trend, but the absolute energy value of those interactions was less than those of the interactions that followed it. Only *KRAS’* ILE^188^ and ATG5’s GLU^248^ strongly contradicted the trend. ILE^183^ of *KRAS* had the least change in binding strength with ATG5 between the complexes. 

### 3.6. Determining Erlotinib Docking Site to EGFR

Of five binding conformations generated by the CB-Dock2 server, erlotinib was determined to be most favorably bound to EGFR, as depicted in Supplementary Figure S1. There were twenty-four EGFR residues which interacted with erlotinib at the most energetically stable binding conformation: VAL^726^, ALA^743^, ILE^744^, LYS^745^, MET^766^, LEU^777^, LEU^788^, ILE^789^, THR^790^, GLN^791^, MET^793^, GLY^796^, CYS^797^, ASP^800^, ASP^837^, ARG^841^, ASN^842^, LEU^844^, THR^854^, ASP^855^, ARG^1068^, TYR^1069^, SER^1070^, and SER^1071^. All these residues are situated in the cytoplasmic region of the protein. CB-Dock2 determined the results were like the crystal structure of EGFR kinase domain L858R mutation in complex with Iressa (pdb: 2ITZ). Iressa (gefitinib) is an EGFR inhibitor that disrupts cell signaling similarly to erlotinib [31]. Notably, the two demonstrate similarity in binding regardless if there is an EGFR mutation. In determining the overall cytotoxicity of the drug class, understanding the interaction behavior is beneficial.

## 4. Discussion

Autophagy expression has been shown to be altered in an array of cancer types but has not been previously tested in colorectal cancer post-erlotinib treatment [32,33]. Previous research has shown that the EGFR inhibitor, erlotinib, can have either positive or negative effects on overall survival for various types of cancers [34,35]. Our findings with cell viability assay demonstrate that erlotinib aids in the killing of CRC cell lines. Interestingly, we also observed increased cell death in erlotinib-treated HKe3 cells (*KRAS*-WT) compared to HCT116 cells (*KRAS*-mut), demonstrating that the *KRAS* mutation diverts the cells to a mechanism that improves the cell viability when compared to wildtype *KRAS*. This is consistent with reports demonstrating that the *KRAS* mutation modulates an important signaling pathway, increasing viability in CRC [36]. The mutation in *KRAS* prevents hydrolysis of its signal-initiating ligand, GTP; thus, promoting cell proliferation may be the mechanism that also improves viability [37].

The effect of erlotinib on autophagy-related protein expression was explored to better understand the mechanism of the drug’s action. Previous experiments have demonstrated that after erlotinib treatment on lung cancer cells, autophagy-related proteins were upregulated [10,38,39]. Interestingly, our data demonstrate a reduction in several autophagy proteins in *KRAS*-WT and *KRAS*-mut cell lines upon erlotinib treatment. The downregulation was more prominent in *KRAS*-mut compared to *KRAS*-WT. Therefore, we propose that the *KRAS* mutation triggers an altered mechanism of cell killing. Thus, this reestablishes the heterogeneity amongst different cancer types. Further, it also indicates that *KRAS* signaling may have different consequences in varying cancer types, which can be correlated to the variation in the expression of cancer-stimulating neoantigens [40,41].

This observation can also be confirmed by the positive correlation found between autophagy and the cell response to stress through cancer metastasis [11]. A separate positive correlation was also discovered between autophagy and stress involving nutrient or growth factor deprivation, hypoxia, reactive oxygen species, DNA damage, protein aggregates, damaged organelles, or intracellular pathogens [42]. Autophagy is known to act as a double-edged sword within the cell. When induction of autophagy cannot salvage the cells, it induces apoptosis, leading to cell killing [43]. Herein, we conclude that autophagy is triggering increased cell killing in the *KRAS* wildtype microenvironment when compared to the *KRAS* mutant. 

The protein expression results from Western blot analysis were then assessed at the level of gene expression by qPCR. The results from the qPCR experiments verified that treatment with erlotinib caused significant downregulation in autophagy gene expression in both *KRAS*-mut and *KRAS*-WT. Furthermore, the downregulation of expression of autophagy-related gene expression was more pronounced in *KRAS*-mut cells compared to *KRAS*-WT cells. Therefore, the diminished gene expression in *KRAS* mutant CRC cells indicates that the process of autophagy might have dual consequences depending on the cancer cell type and, thus, plays a crucial role in overall cell survival.

CRC patient mRNA expression databases were analyzed using GEPIA2 to assess correlation analyses between *KRAS* and the five autophagy proteins MAP1LC3A, ATG5, ATG10, ATG13, and ATG14. Xena was also used to assess autophagy-related gene expression in cancer patients for the same proteins. In the absence of erlotinib treatment, ATG10 was found to be upregulated, while MAP1LC3A, ATG5, ATG13, and ATG14 were significantly downregulated (Figure 4). Using GEPIA2, a significant positive correlation was found between the gene expression of all five autophagy proteins (MAP1LC3A, ATG5, ATG10, ATG13, and ATG14) and *KRAS* gene expression. Xena analysis demonstrated that the autophagy mRNA genes MAP1LC3A, ATG5, ATG13, and ATG14 were more downregulated in *KRAS*-mut samples compared to *KRAS*-WT, which is similar to our observations. In contrast, ATG10 mRNA expression was higher in *KRAS*-mut compared to *KRAS*-WT. These results show that before treatment with erlotinib, there was already a significant difference in the expression of the autophagy genes between *KRAS*-mut and *KRAS*-WT. Therefore, erlotinib treatment results in further downregulation of autophagy. This also confirms that there is a crosstalk between *KRAS* and autophagy proteins. 

Our molecular dynamics simulation of ATG5 with *KRAS*-WT and the G13D mutant supports our claim that there is a connection between *KRAS* and autophagic proteins and that the mutation impacts their interaction. ATG5 was used in simulations with *KRAS*-WT due to its significant contribution to autophagy expression. Furthermore, we constructed a blueprint of their interactions, analyzing the structural and kinetic components of the WT and G13D complexes. Taken together, this suggests that autophagy downregulation serves to positively impact *KRAS*-mut patient outcomes in CRC.

It is common for patients to develop resistance to erlotinib as it is a competitive inhibitor [17]. A dual treatment of erlotinib alongside other approved therapies may be beneficial. Higher expression of autophagy promotes erlotinib action in increased cell death in *KRAS*-WT (HKe3) compared to *KRAS*-mut (HCT116 cells). Previous experiments in lung cancer demonstrated that erlotinib, in combination with other drugs such as cisplatin and chloroquine, can overcome drug resistance [18,44]. Chloroquine has been tested thoroughly to act as an inhibitor of autophagy, which provides evidence that erlotinib treatment results in a similar outcome [44]. As of yet, there has been little research testing erlotinib combination therapies in colorectal cancer cells, which could solve potential resistance issues in CRC.

## Figures and Tables

**Table 1:** Primer Sequences.

Primer Name	Forward	Reverse
ULK1	CACGCCACATAACAGACAAAAATAC	ACAAGGTGAGAATAAAGCCATCAAG
ATG5	TTGGACGAATTCCAACTTGTTTC	ATTTCAGTGGTGTGCCTTCATATTC
MAP1LC3B	AGCAGCATCCAACCAAAATC	CTGTGTCCGTTCACCAACAG
BECN1	ACAACAAGTTTGACCATGCAA	TTCAATCTTGCCTTTCTCCACAT
GAPDH	CTTTTGCGTCGCCAG	TTGATGGCAACAATATCC

**Table 2:** Fold change (FC) difference in cell viability levels between HCT116 and HKe3.

Hours of Treatment	Erlotinib Treatment	HCT116	HKe3	*p*-Value
9	20 μM	0.91	1.00	0.026
	35 μM	0.89	0.76	0.013
24	20 μM	0.88	0.66	0.017
	35 μM	0.73	0.62	0.200
48	20 μM	0.75	0.61	0.049
	35 μM	0.68	0.47	0.010

**Table 3:** Fold change (FC) difference in protein levels of autophagy-related genes in HCT116 and HKe3.

20 μM of Erlotinib Treatment Protein Fold Change			
Autophagy Protein	HCT116	HKe3	*p*-Value (HCT116 vs. HKe3)
ATG5	0.60	0.99	0.023
ULK1	0.73	0.92	0.003
Beclin-1	0.43	0.91	0.039
LC3A	0.40	0.80	0.019

**Table 4:** Fold change (FC) difference in mRNA levels of autophagy-related genes between HCT116 and HKe3.

20 μM of Erlotinib Treatment			
Autophagy mRNA	HCT116	HKe3	*p*-Value (HCT116 vs. HKe3)
ATG5	0.82	1.00	0.019
ULK1	0.87	0.98	0.029
BECN1	0.74	0.98	0.019
MAP1LC3B	0.63	0.87	0.010

**Table 5:** Correlation analysis of *KRAS* and autophagic mRNA in COADREAD (colon adenocarcinoma) and normal tissue without erlotinib treatment.

mRNA Correlation Analysis (R)		
mRNA	COADREAD	Normal Tissue
*MAP1LC3B*	0.84	0.96
*ATG5*	0.87	0.94
*ATG10*	0.85	0.93
*ATG13*	0.86	0.94
*ATG14*	0.85	0.94

**Table 6:** mRNA expression analysis between autophagy mRNA in cancer patients without erlotinib treatment. An empty data point represents a non-significant expression.

mRNA Expression Change in CRC	
mRNA	Cancer Patients
*MAP1LC3B*	76%
*ATG5*	81%
*ATG10*	----
*ATG13*	85%
*ATG14*	86%

**Table 7:** Total complex and delta energy of WT and G13D complexes with ATG5 (kcal/mol).

	WT-ATG5	G13D-ATG5
Total Complex Mean	−3142.8	−3119.2
Total Complex STD	20.9	21.5
Delta Mean	−48.4	−47.7
Delta STD	5.3	6.1

## Data Availability

Data are contained within the article and supplementary materials.

## Supplementary Materials

The following supporting information can be downloaded at https://www.mdpi.com/article/10.3390/cimb46070447/s1, Figure S1: Erlotinib Docking Site to EGFR.
