# Supplementary figures and images for: Erlotinib Treatment in Colorectal Cancer Suppresses Autophagy Based on KRAS Mutation
Source: Curr Issues Mol Biol. 2024 Jul 16;46(7):7530–47. doi: 10.3390/cimb46070447 (PMC11276370; doi:10.3390/cimb46070447)

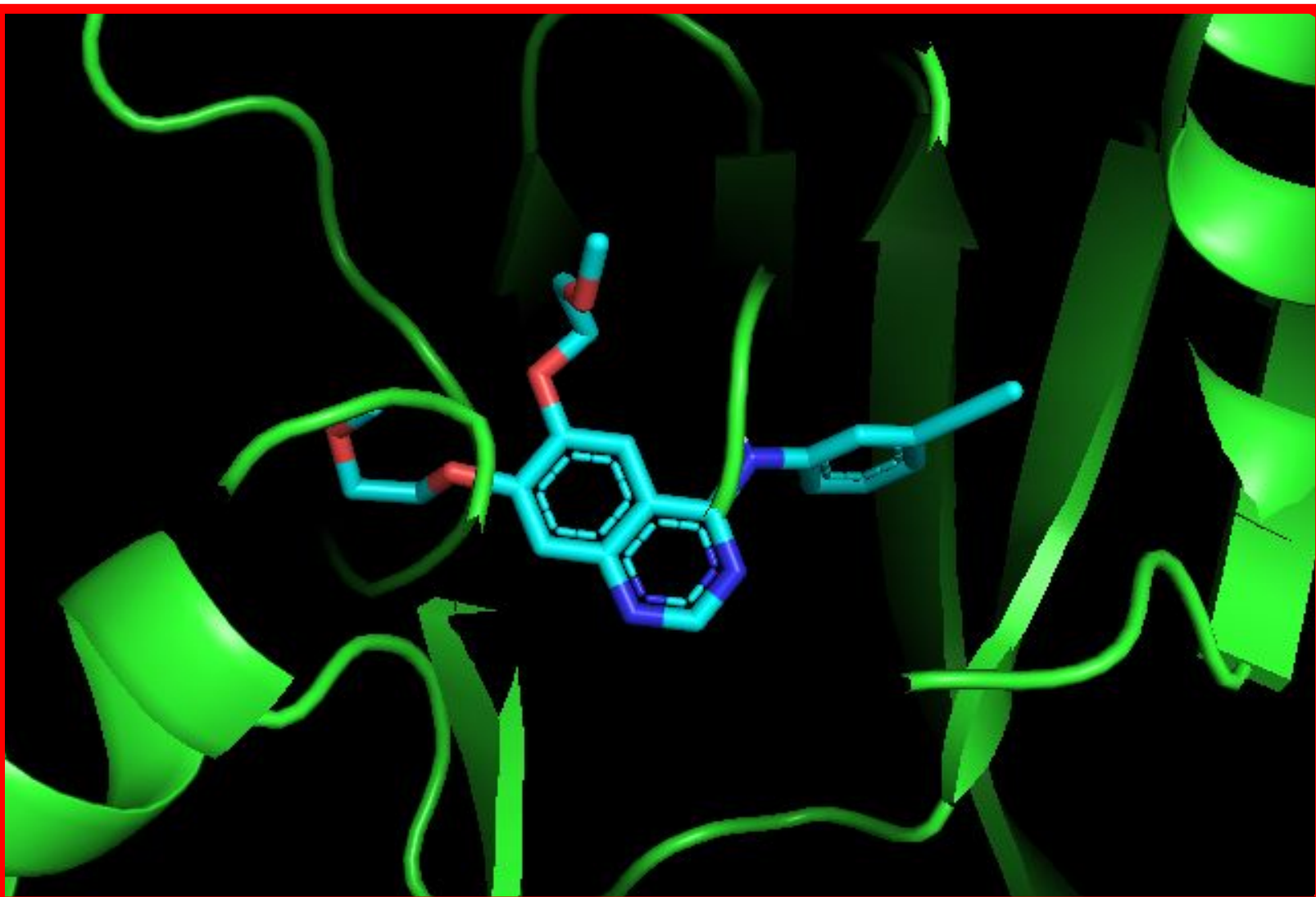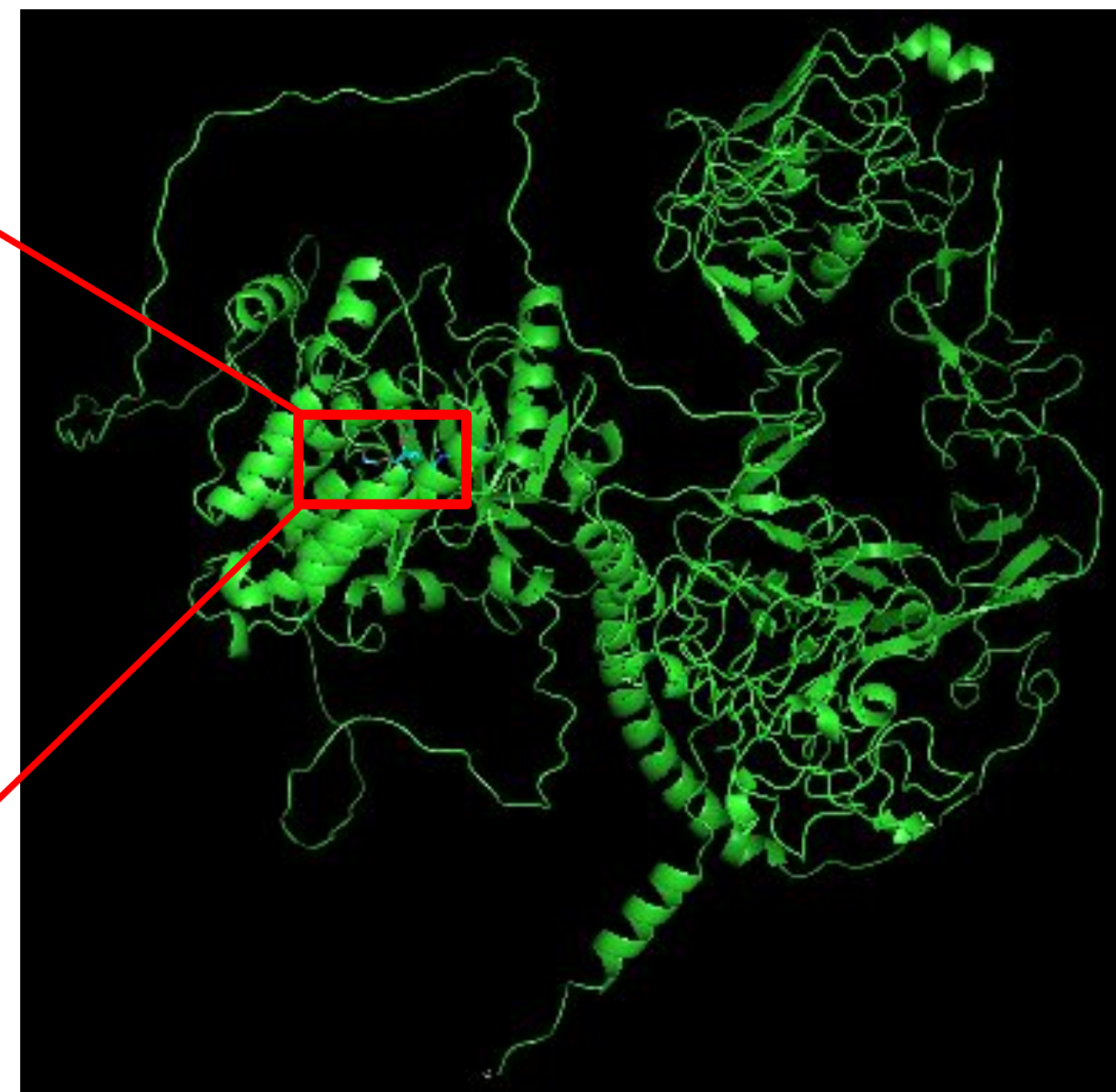

Supplement: Supplementary file 1 [file cimb-46-00447-s001.zip › cimb-3083524-supplementary.pdf]
